# Supplementary material for: Supraphysiological activation of TAK1 promotes skeletal muscle growth and mitigates neurogenic atrophy
Source: Nat Commun. 2022 Apr 22;13:2201. doi: 10.1038/s41467-022-29752-0 (PMC9033787; doi:10.1038/s41467-022-29752-0)
Supplement: Supplementary file 1 — Supplementary Information [file 41467_2022_29752_MOESM1_ESM.pdf]

Supplementary Data File

Supraphysiological activation of TAK1 promotes skeletal muscle growth and mitigates neurogenic atrophy

By

Anirban Roy[1] and Ashok Kumar[1*]

[1]Department of Pharmacological and Pharmaceutical Sciences, University of Houston College of Pharmacy, Houston, TX 77204, USA

***Corresponding author:**

Ashok Kumar, Ph.D.
Department of Pharmacological and Pharmaceutical Sciences
Health Building 2, Room 5012
College of Pharmacy
University of Houston
4849 Calhoun Road
Houston, TX 77204-1217

Phone: 713-743-3376
Email: akumar43@Central.UH.EDU

This file contains:

Supplementary Figures 1-9

Supplementary Table 1 and 2.

Supplementary Figure 1

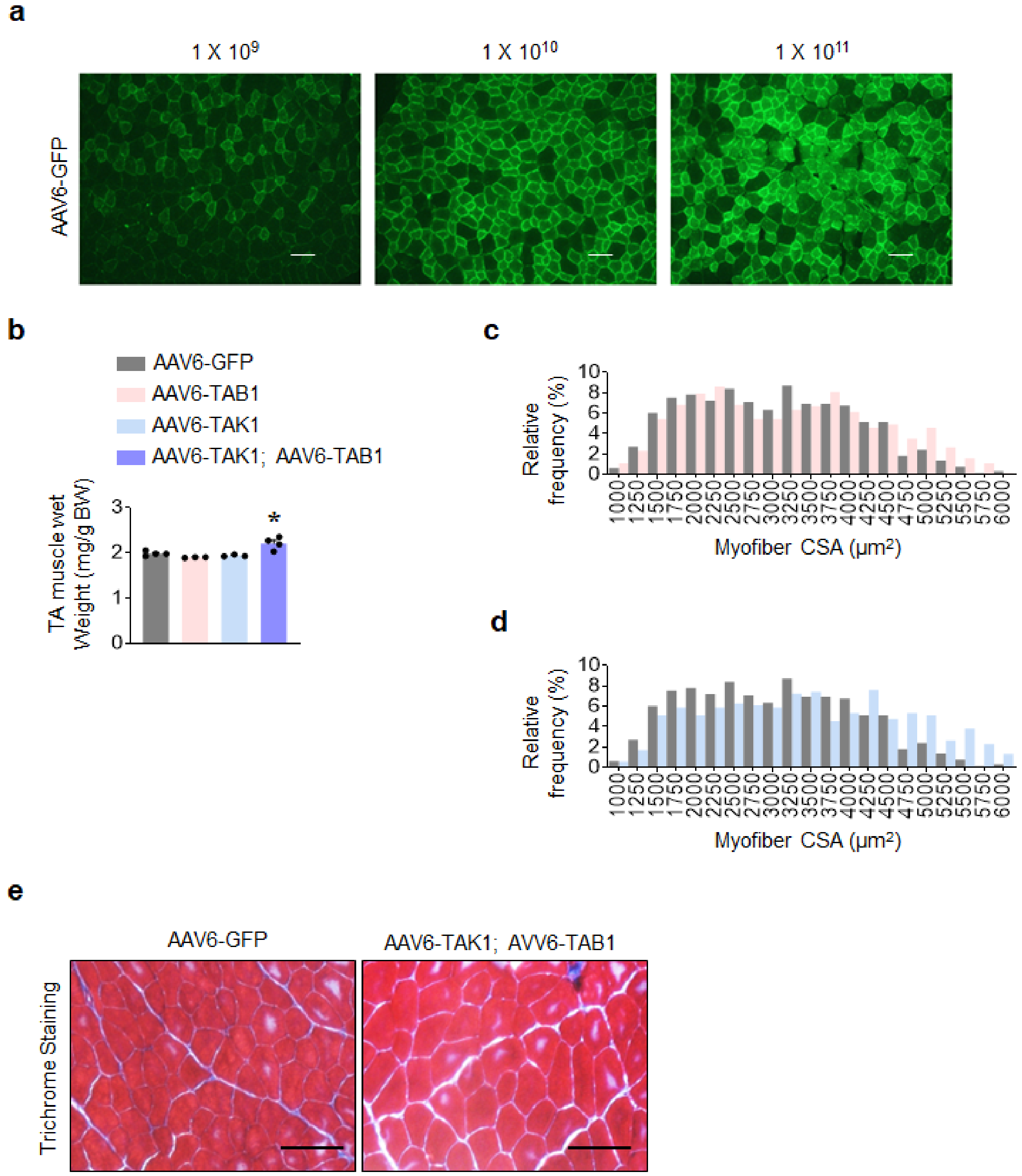

Supplementary Figure 1. Effect of overexpression of TAK1, TAB1, or combination of TAK1 and TAB1 on muscle growth and fibrosis. (a) TA muscles of 3-month old C57BL/6

mice were injected with AAV6-GFP vector at indicated doses. After 5 days, the mice were sacrificed and TA muscle was isolated. Finally, transverse TA muscle sections were generated and analyzed for GFP expression using a fluorescence microscope. Representative photomicrographs are presented here. Scale bar, 100 μm. **(b)** Left side TA muscle of C57BL/6 mice was given intramuscular injection of AAV6-TAB1 (2.5 x 10^{10} vg), AAV6-TAK1 (2.5 x 10^{10} vg), or a combination of AAV6-TAB1 (1.25 x 10^{10} vg) and AAV6-TAK1 (1.25 x 10^{10} vg) while the contralateral right TA muscle was injected with AAV6-GFP (2.5 x 10^{10} vg) particles. After 28 days, the mice were euthanized and the TA muscles were harvested and analyzed. Average TA muscle weight normalized with body weight (BW) of mice. N=3-4 in each group. Data presented as mean ± SEM. *$p<0.05$, values significantly different from contralateral AAV6-GFP alone injected TA muscle by one-way ANOVA followed by Tukey's multiple comparison test. **(c)** Relative frequency distribution of myofiber cross sectional area (CSA) in TA muscle injected with AAV6-GFP or AAV6-TAB1 alone. **(d)** Relative frequency distribution of myofiber CSA in TA muscle injected with AAV6-GFP or AAV6-TAK1 alone. **(e)** TA muscle sections made were stained with Trichrome Masson's staining. Representative photomicrographs are presented here. Scale bar, 100 μm.

Supplementary Figure 2

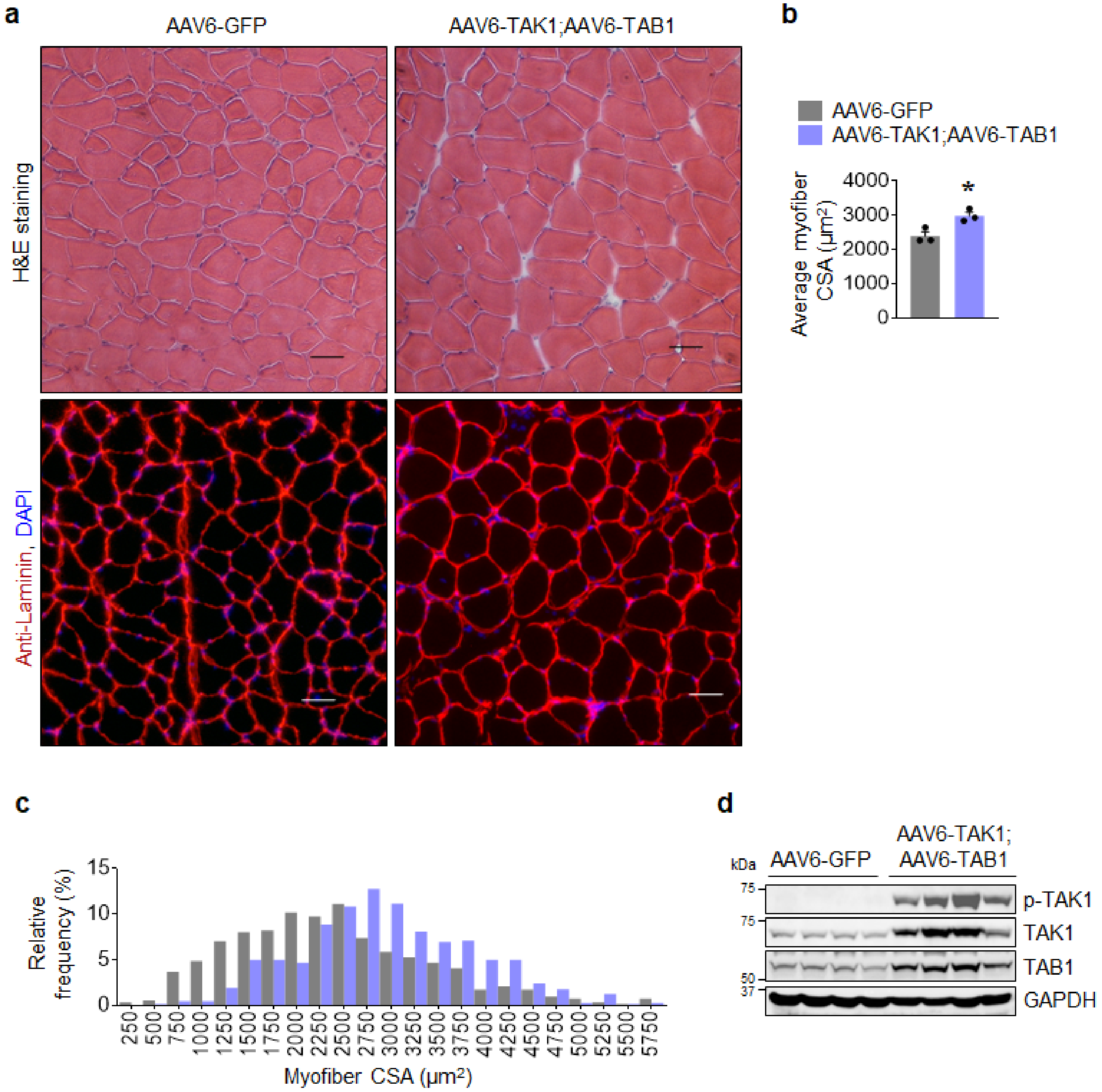

Supplementary Figure 2. Forced activation of TAK1 induces myofiber hypertrophy in gastrocnemius muscle. Left side GA muscle of C57BL/6 mice was given intramuscular injection of a combination of AAV6-TAB1 (1.25 x 10^{10} vg) and AAV6-TAK1 (1.25 x 10^{10} vg) while the contralateral right TA muscle was injected with AAV6-GFP (2.5 x 10^{10} vg) particles. After 28 days, the mice were euthanized and the GA muscle was harvested and analyzed. **(a)**

Representative photomicrographs of GA muscle transverse sections after H&E staining or anti-laminin and DAPI staining. Scale bar, 50 μm. **(b)** Average myofiber cross-sectional area (CSA) in GA muscle of mice injected with AAV6-GFP or a combination of AAV-TAK1 and AAV-TAB1. n=3 mice in each group. **(c)** Relative frequency distribution of myofiber CSA in GA muscle injected with AAV6-GFP or combination of AAV6-TAK1 and AAV6-TAB1. n= 4 mice in each group. (**d**) Immunoblots presented here demonstrate levels of p-TAK1 and total TAK1, TAB1, and GAPDH protein in GA muscle of mice after 28 days of intramuscular injection of AAVs. N=4 in each group. Results are presented as mean ± SEM. *p<0.05, values significantly different from contralateral GA muscle injected with AAV6-GFP alone by unpaired *t*-test.

Supplementary Figure 3

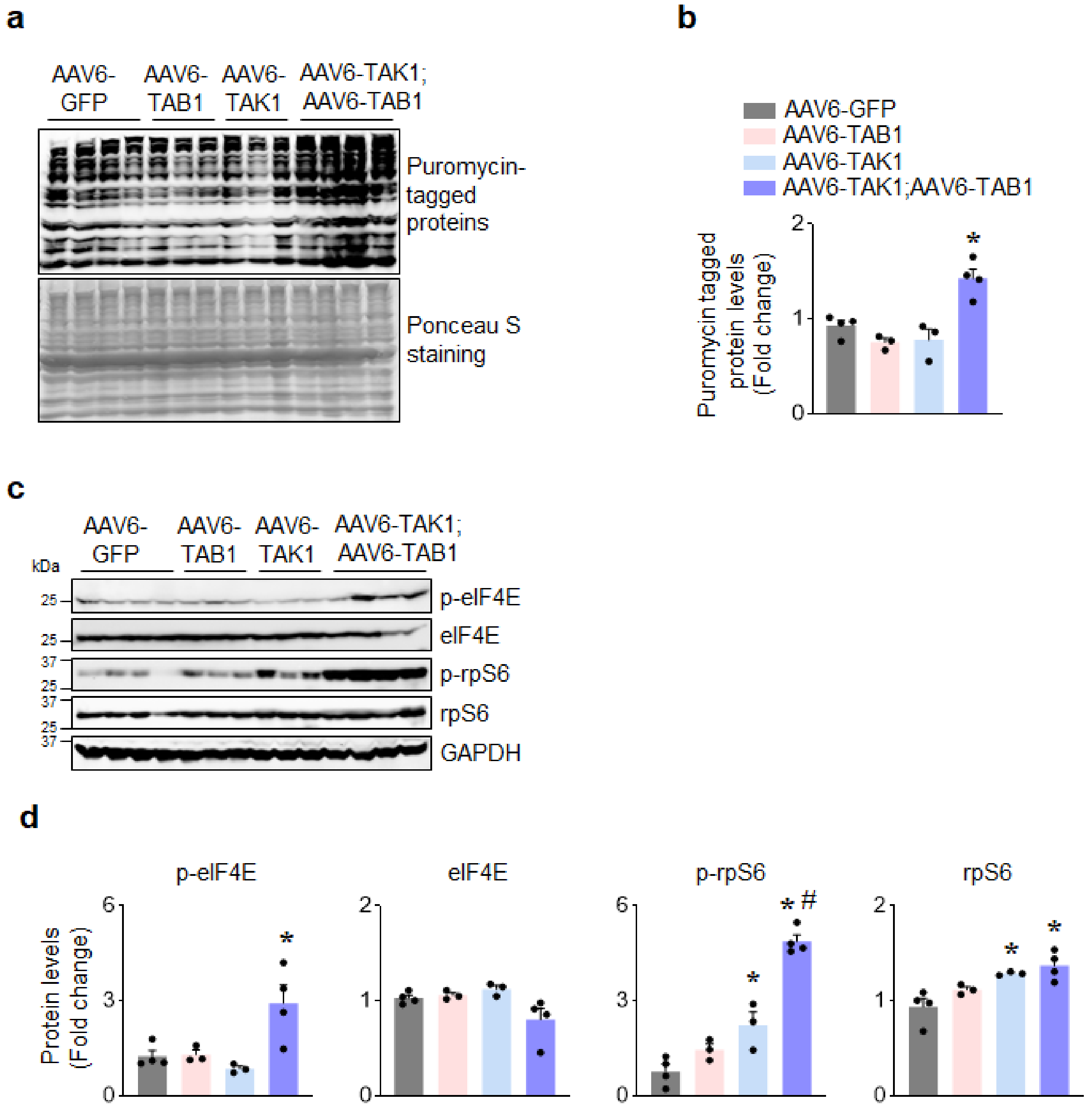

Supplementary Figure 3. Forced activation of TAK1 induces protein synthesis in skeletal muscle of mice. Left side TA muscle of C57BL/6 mice was given intramuscular injection of AAV6-TAB1 (2.5 x 10^{10} vg), AAV6-TAK1 (2.5 x 10^{10} vg), or a combination of AAV6-TAB1 (1.25 x 10^{10} vg) and AAV6-TAK1 (1.25 x 10^{10} vg) while the contralateral right TA muscle was injected with AAV6-GFP (2.5 x 10^{10} vg) particles. At day 28, the mice were given

intraperitoneal injection of puromycin and 30 min later the mice were euthanized and TA muscle was harvested for analysis. **(a)** Representative immunoblot **(b)** densitometry analysis of puromycin-tagged protein in TA muscle of mice injected with AAV6-GFP, AAV6-TAB1, AAV6-TAK1 or combination of AAV6-TAB1 and AAV6-TAK1. **(c)** Representative immunoblots, and **(d)** densitometry analysis of levels of p-eIF4E, eIF4E, p-rpS6, and rpS6 in protein in TA muscle of mice injected with AAV6-GFP, AAV6-TAB1, AAV6-TAK1, or combination of AAV6-TAB1 and AAV6-TAK1. N=3-4 in each group. Results are presented as mean ± SEM. *p<0.05, values significantly different from TA muscle injected with AAV6-GFP alone by unpaired *t*-test. #p<0.05, values significantly different from TA muscle injected with AAV6-TAK1 alone by unpaired t-test.

Supplementary Figure 4

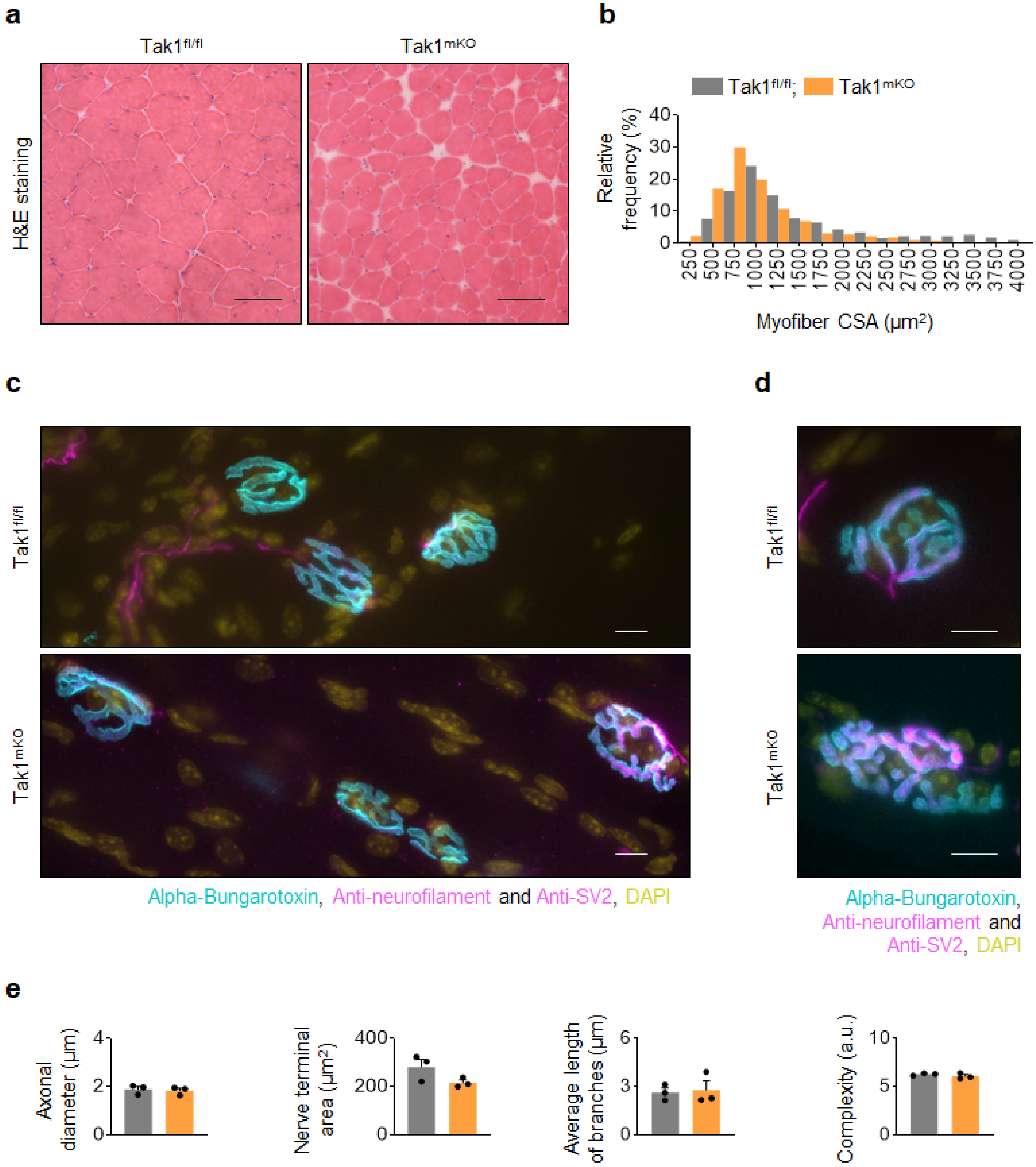

Supplementary Figure 4. Role of TAK1 in regulation of muscle mass and NMJs.

Littermate Tak1$^{fl/fl}$ and Tak1^{mKO} mice were administered with tamoxifen i.p. (75mg/kg

bodyweight) for 4 days and then fed with tamoxifen containing diet for 24 days to delete Tak1 gene. **(a)** Representative photomicrograph of H&E-stained TA muscle transverse sections. Scale bar, 50 μm. **(b)** Percent relative frequency of myofiber CSA in TA muscle of $Tak1^{fl/fl}$ and $Tak1^{mKO}$ mice. Representative immunofluorescence images of NMJ at **(c)** lower and **(d)** higher magnification in $Tak1^{fl/fl}$ and $Tak1^{mKO}$ mice. Scale bar, 10 μm. **(e)** Presynaptic parameters of NMJ analyzed using ImageJ software. n=3 mice in each group. Results are presented as mean ± SEM. No statistically significantly differences were observed between $Tak1^{fl/fl}$ and $Tak1^{mKO}$ mice by unpaired t-test.

Supplementary Figure 5

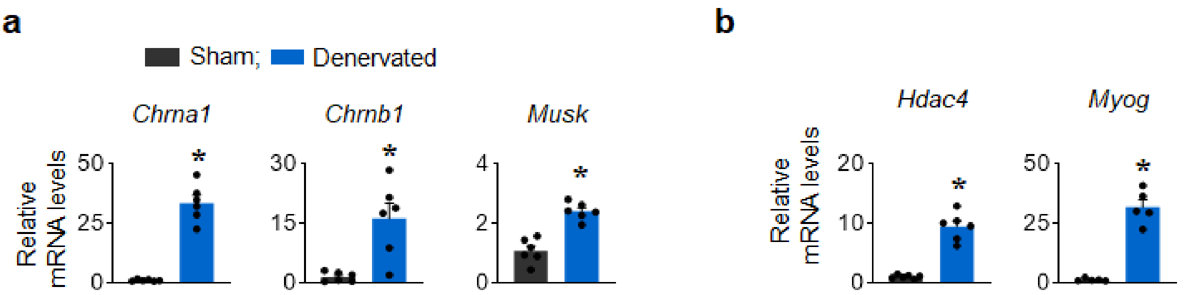

Supplementary Figure 5. Increased expression of NMJ components in denervated muscle. Adult C57BL6 mice were subjected sham or denervation surgery. After 7 days, GA muscle was isolated and analyzed by qPCR. Relative mRNA levels of **(a)** *Chrna1* (AChRα), *Chrnb1* (AChRβ), *Musk* and **(b)** *Hdac4* and *Myog* in sham and denervated muscle GA muscle. n=4-6 in each group. Results are presented as mean ± SEM. *p<0.05, values significantly different from undenervated (sham-operated) muscle by unpaired *t*-test.

Supplementary Figure 6

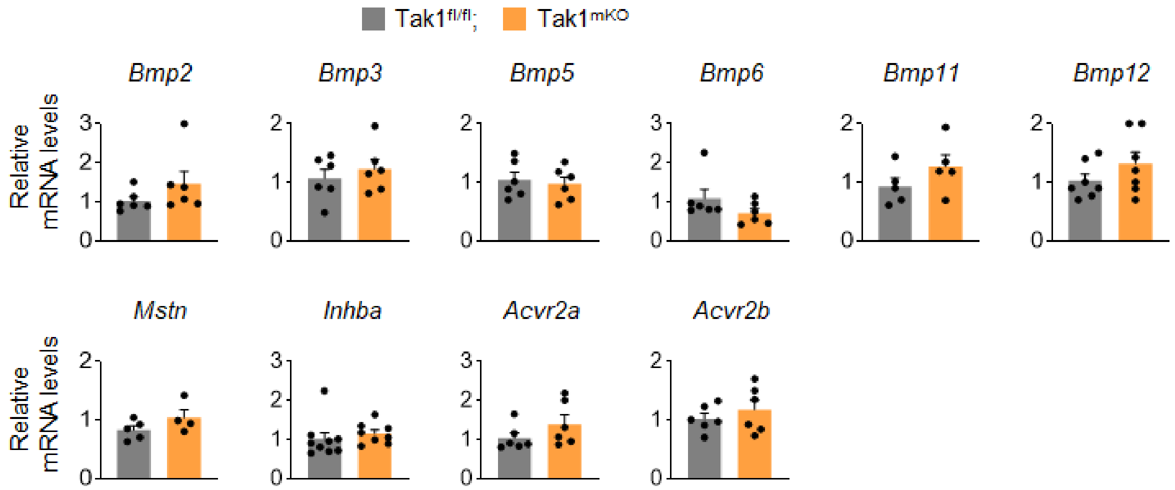

Supplementary Figure 6. Effect of inactivation of TAK1 on gene expression of BMP and TGFβ subfamily molecules. Relative mRNA levels of Bmp2, Bmp3, Bmp5, Bmp6, Bmp11, Bmp12, Mstn (myostatin), *Inhba* (inhibin beta A), *Acvr2a* receptor and *Acvr2b* receptor in GA muscle of Tak1$^{fl/fl}$ and Tak1^{mKO} mice assayed by qPCR. n=5-9 per group. Data presented here are mean ±SEM. No statistically significantly differences were observed between Tak1$^{fl/fl}$ and Tak1^{mKO} mice by unpaired t-test.

Supplementary Figure 7

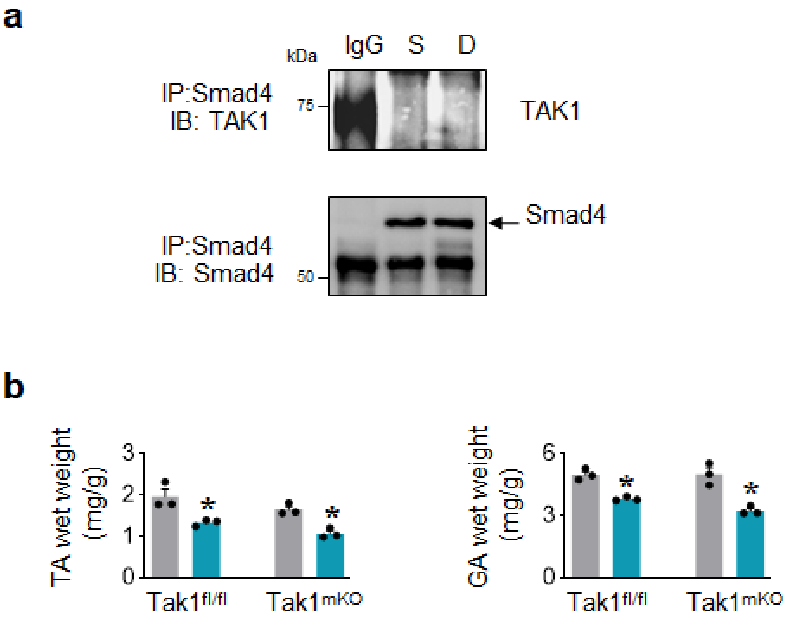

Supplementary Figure 7. TAK1 regulates Smad signaling in denervated muscle. C57BL/6 mice were subjected to sham or denervation surgery for 7d followed by isolation of hind limb muscle and biochemical analysis. **(a)** Immunoblots after immunoprecipitation of GA muscle extracts with Smad4 antibody followed by immunoblotting with TAK1 or Smad4 antibody. (**b**) $Tak1^{fl/fl}$ and $Tak1^{mKO}$ mice were administered with tamoxifen i.p. (75mg/kg bodyweight) for 4 days and were fed tamoxifen containing diet thereafter. At day 7 after first tamoxifen injection, the left hind leg was denervated by transecting the sciatic nerve whereas sham surgery was performed on the right leg. After 7 days, hind limb muscles were harvested and analyzed. Wet weight of sham-operated and 7d-denervated TA, and GA muscle normalized with body weight of $Tak1^{fl/fl}$ and $Tak1^{mKO}$ mice. Data presented here are mean ±SEM. *$p<0.05$, values significantly different from corresponding sham-operated muscle by two-way ANOVA followed by Tukey’s multiple comparison test.

Supplementary Figure 8

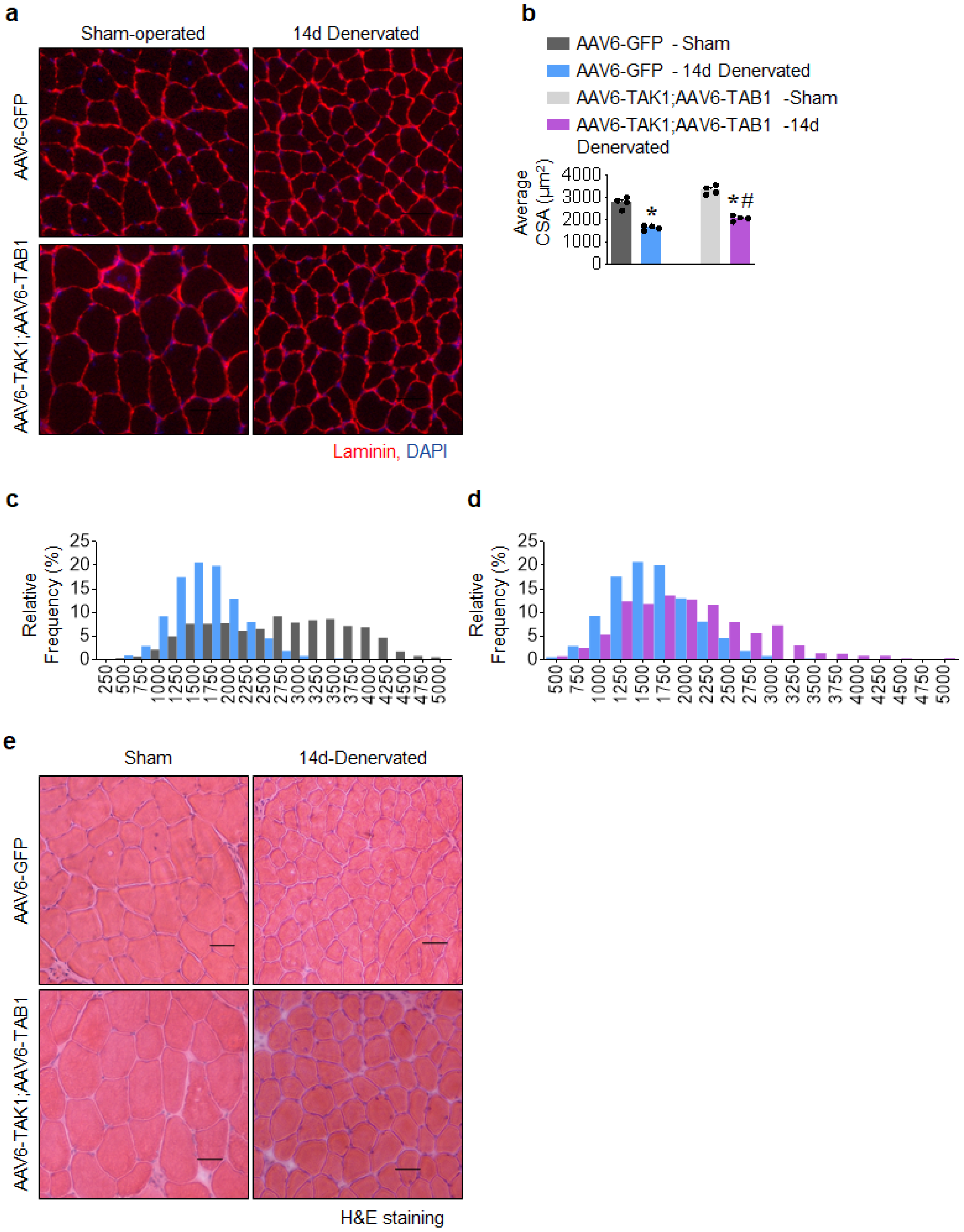

Supplementary Figure 8. Forced activation of TAK1 mitigates denervation-induced muscle atrophy. Both side TA muscle of adult wild-type mice were given intramuscular

injection of AAV-GFP (2.5 X 10^{10} vg) or a combination of AAV-TAK1 (1.25 X 10^{10} vg) and AAV-TAB1 (1.25 X 10^{10} vg) particles. After 14 days, the left hind muscles of the mice were denervated whereas right side was sham operated. On day 14 post denervation, the mice were euthanized and TA muscles were harvested and analyzed. **(a)** Representative photomicrographs of TA muscle sections after staining with anti-laminin and DAPI. Scale bar, 50 □m. **(b)** Average myofiber cross sectional area (CSA) in sham and 14d-denervated TA muscle injected with AAV6-GFP or combination of AAV6-TAK1 and AAV6-TAB1. **(c)** Relative frequency distribution of myofiber CSA expressed as percentage in sham and 14d-denervated TA muscles injected with AAV6-GFP. **(d)** Relative frequency distribution of myofiber CSA in 14d-denervated TA muscles injected with AAV6-GFP compared with 14d-denervated TA muscle co-injected with AAV6-TAK1 and AAV6-TAB1. (e) Representative H&E-stained images of sham and 14d-denervated TA muscle sections injected with AAV6-GFP or a combination of AAV6-TAK1 and AAV6-TAB1. N=4 in each group. Data are presented as mean ±SEM and was analyzed by two-way ANOVA followed by Tukey's multiple comparison test. *$p<0.05$, values significantly different from corresponding control sham-operated TA muscle. $^{\#}p<0.05$, values significantly different from 14d-denervated TA muscle injected with AAV6-GFP.

Supplementary Figure 9

Figure 1a

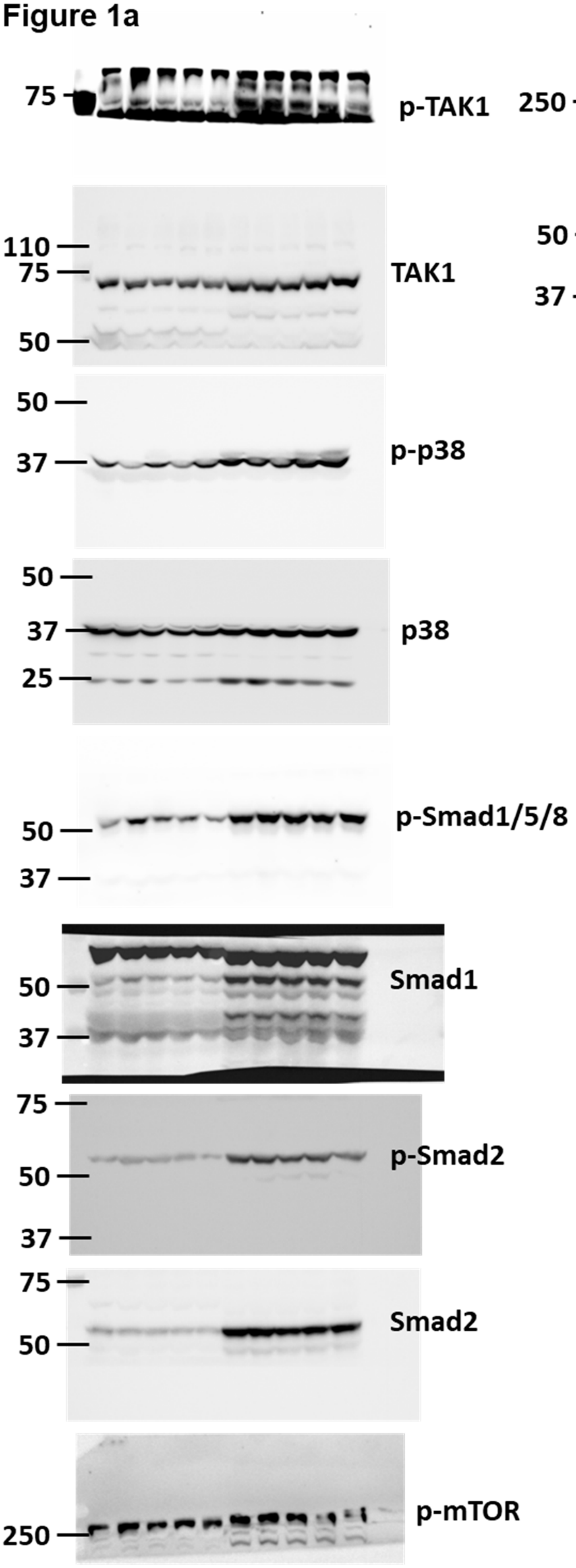

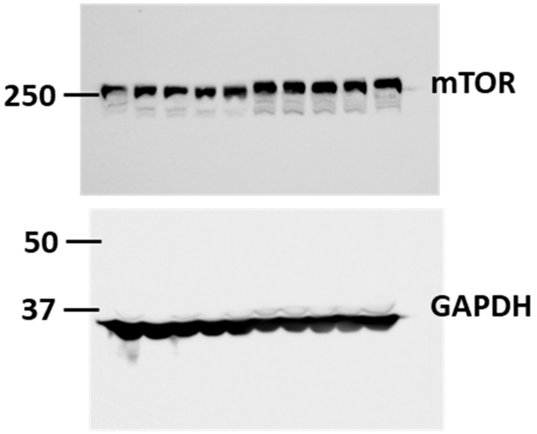

Figure 1f

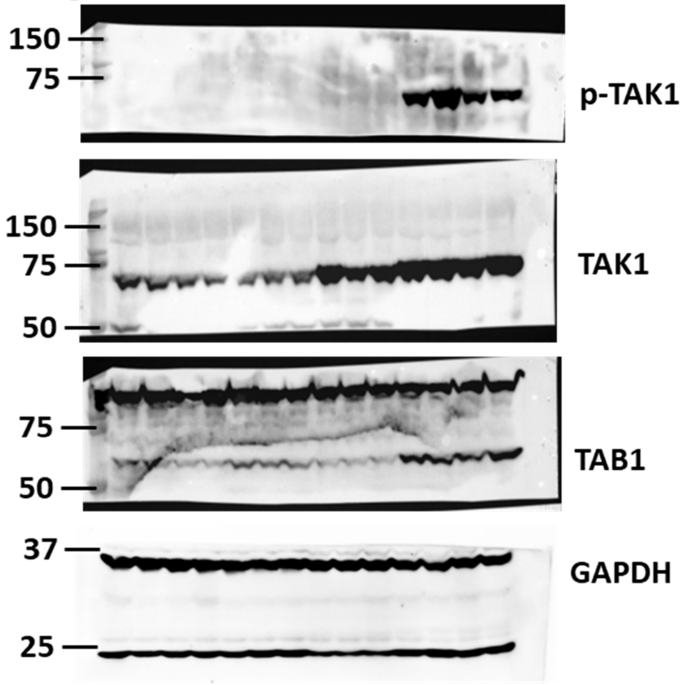

Supplementary Figure 9 contd.

Figure 2a

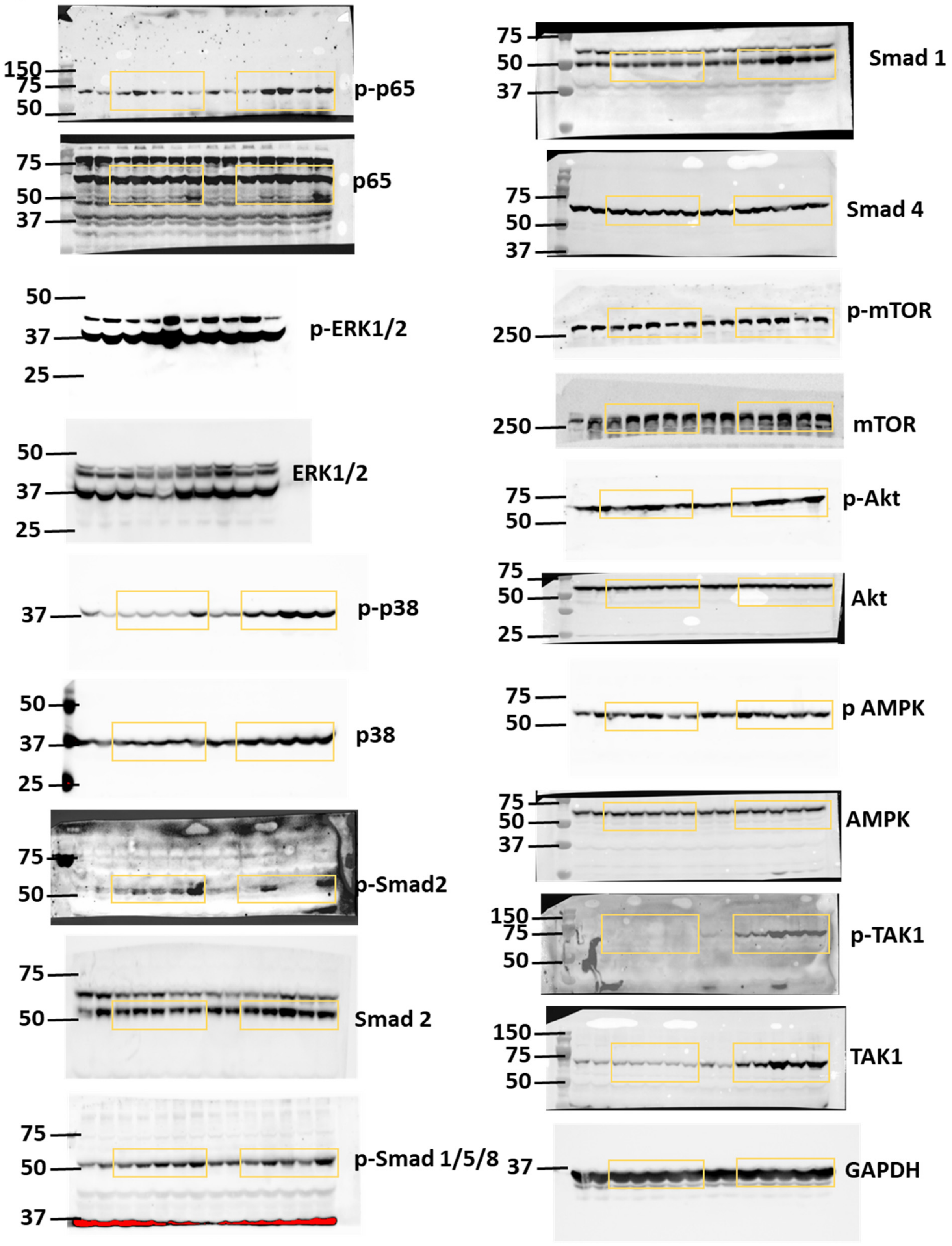

Supplementary Figure 9 contd.

Figure 2b

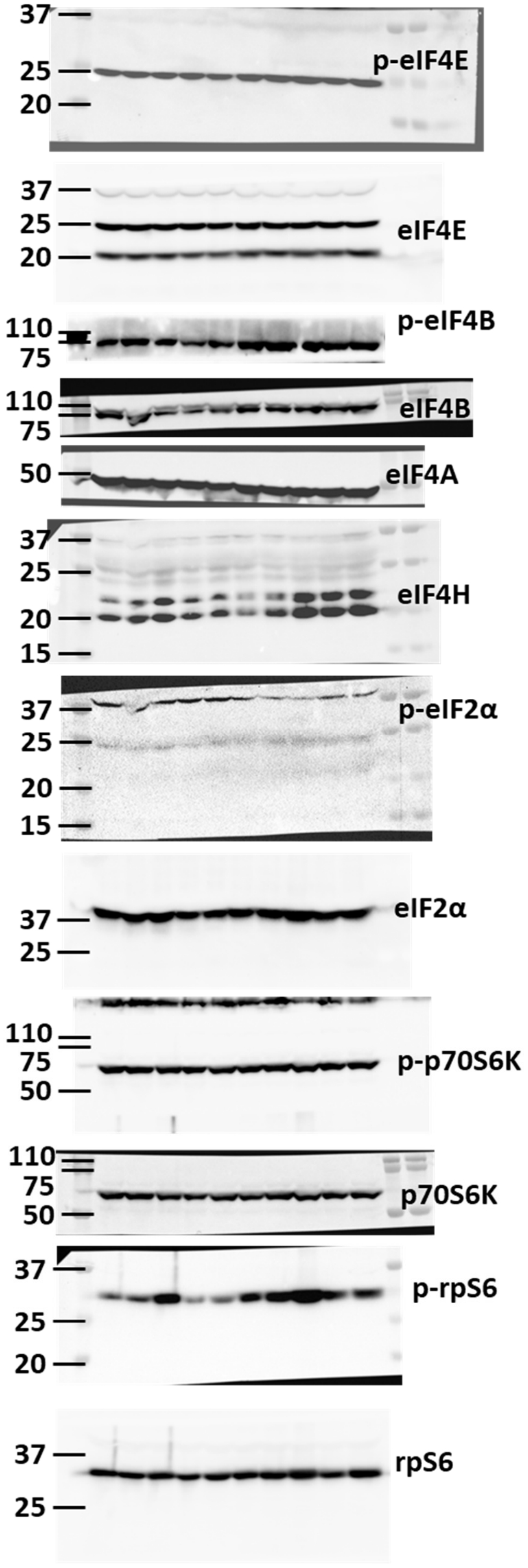

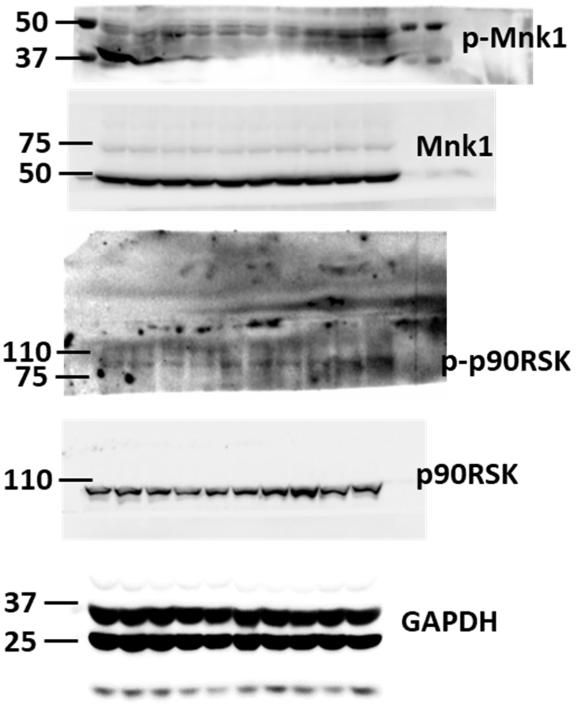

Figure 3b.

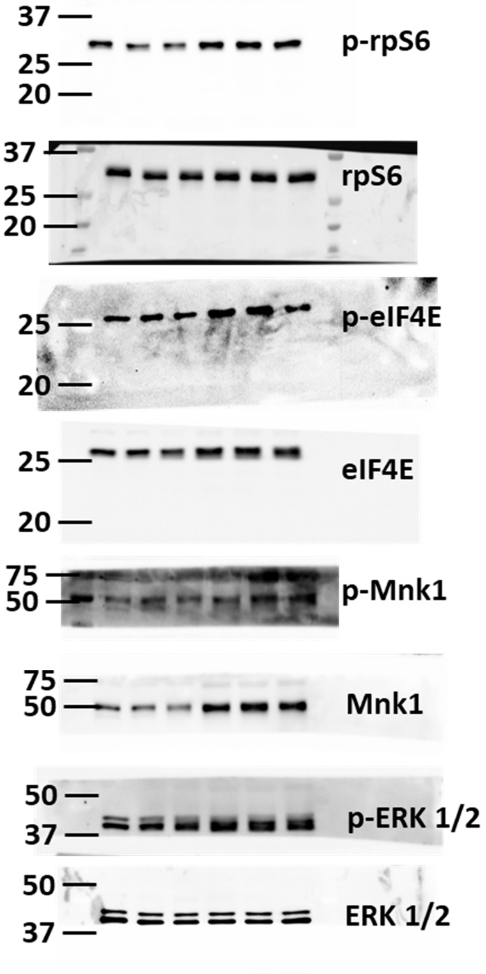

Supplementary Figure 9 contd.

Figure 3b contd.

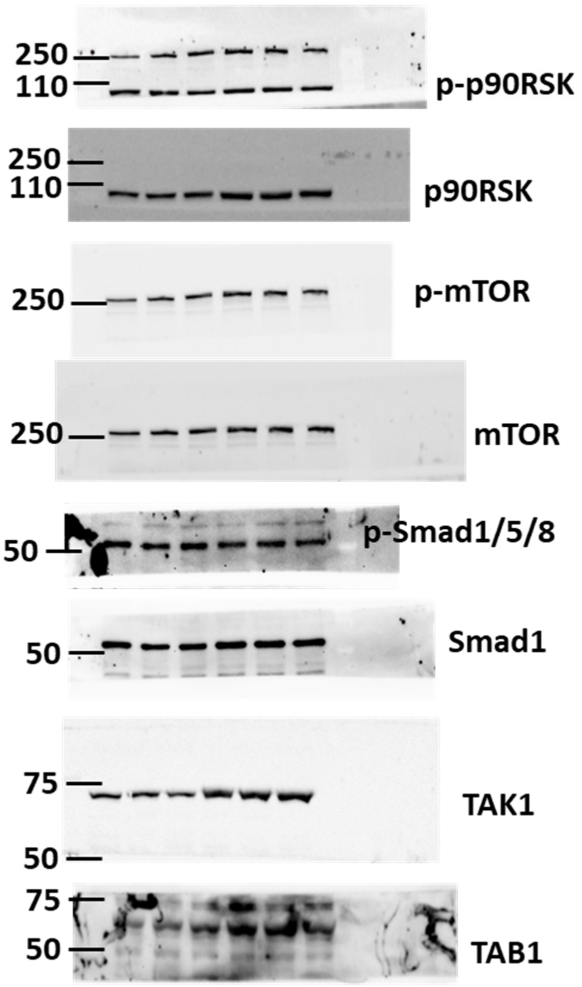

Figure 3f.

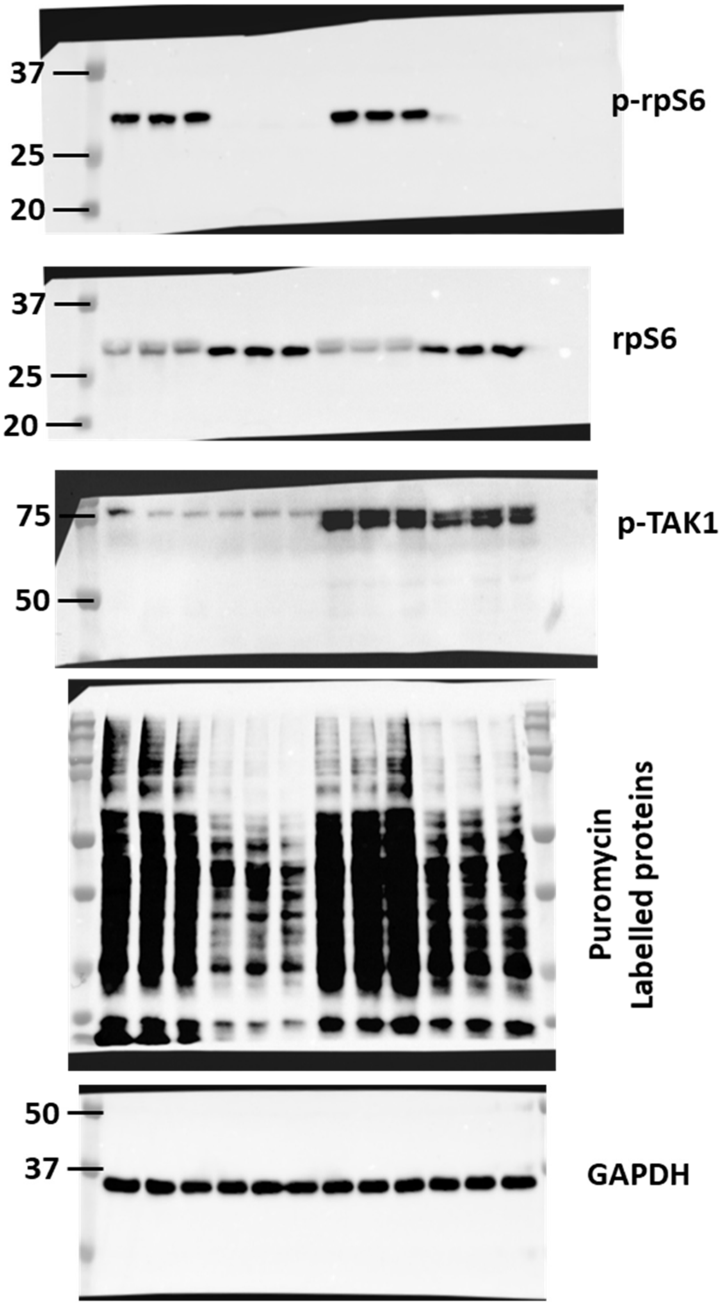

Figure 3d.

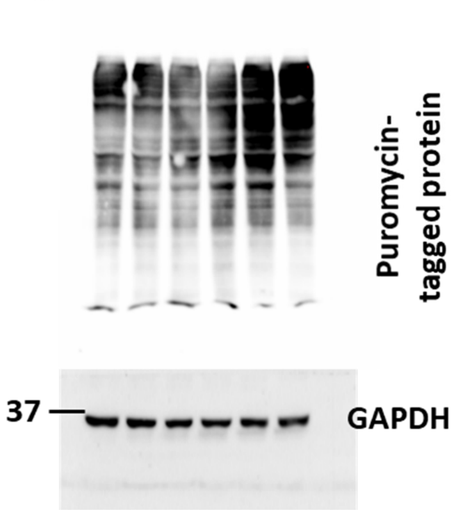

Supplementary Figure 9 contd.

Figure 4e.

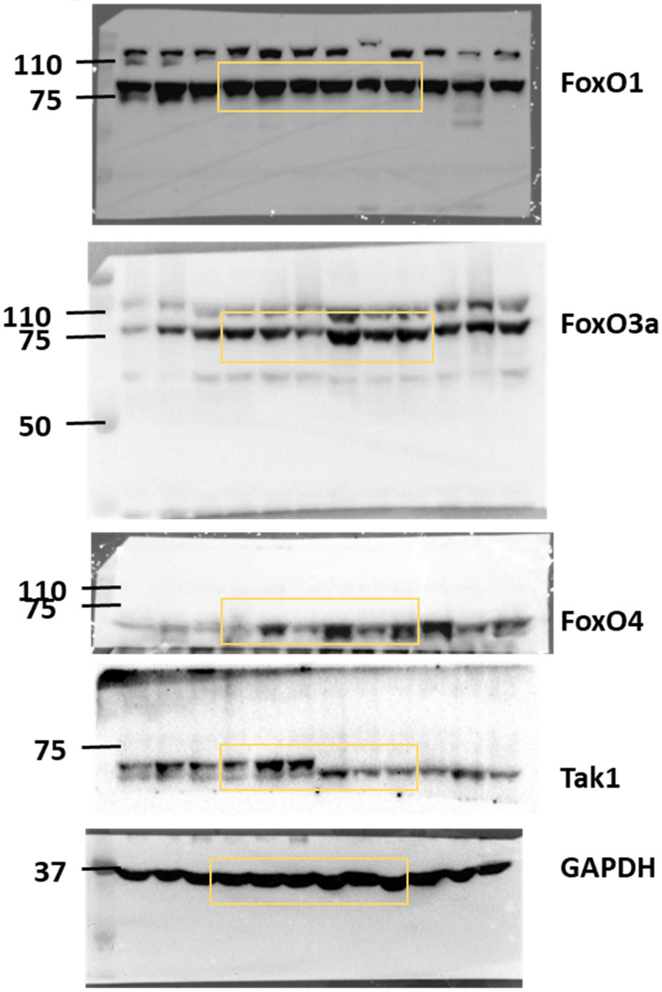

Figure 4h.

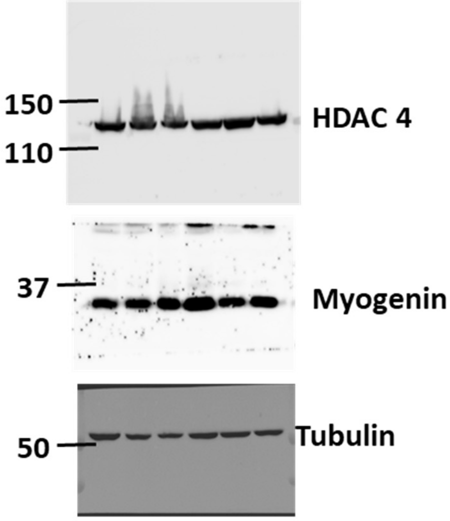

Figure 5a.

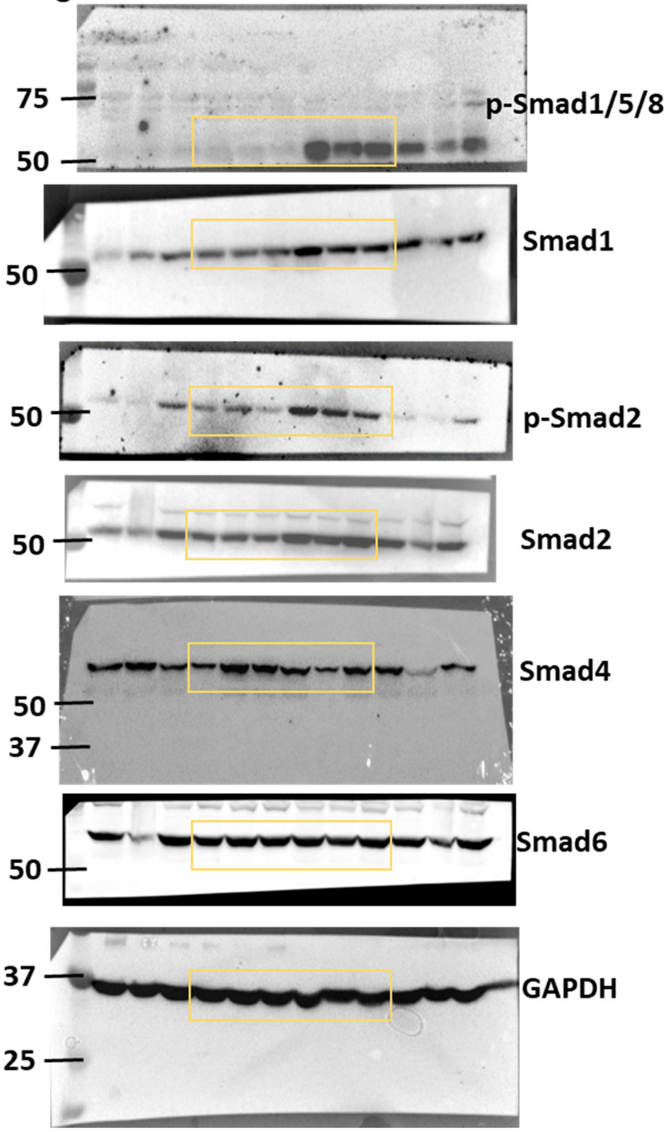

Supplementary Figure 9 contd.

Figure 6a.

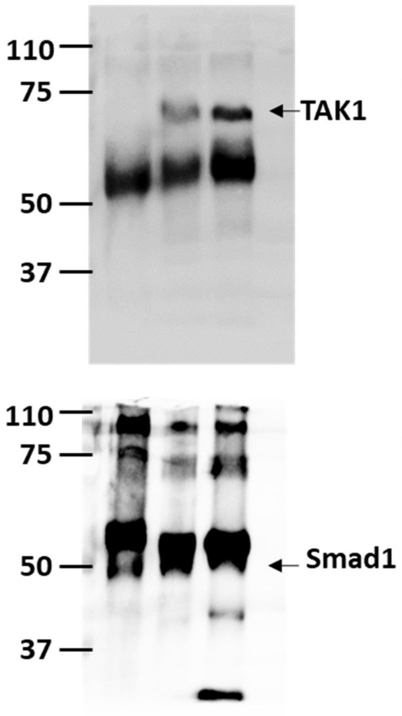

Figure 6c.

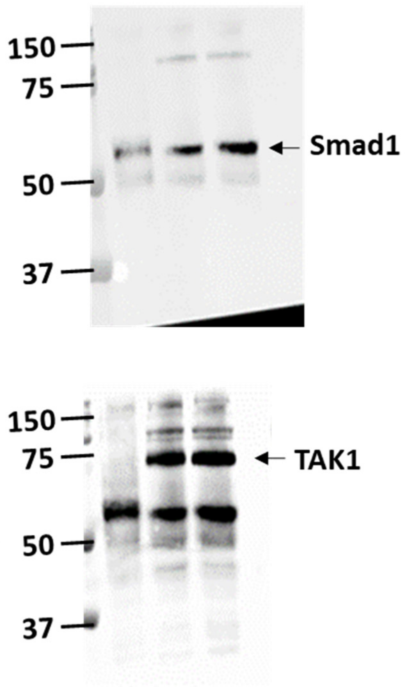

Figure 6d.

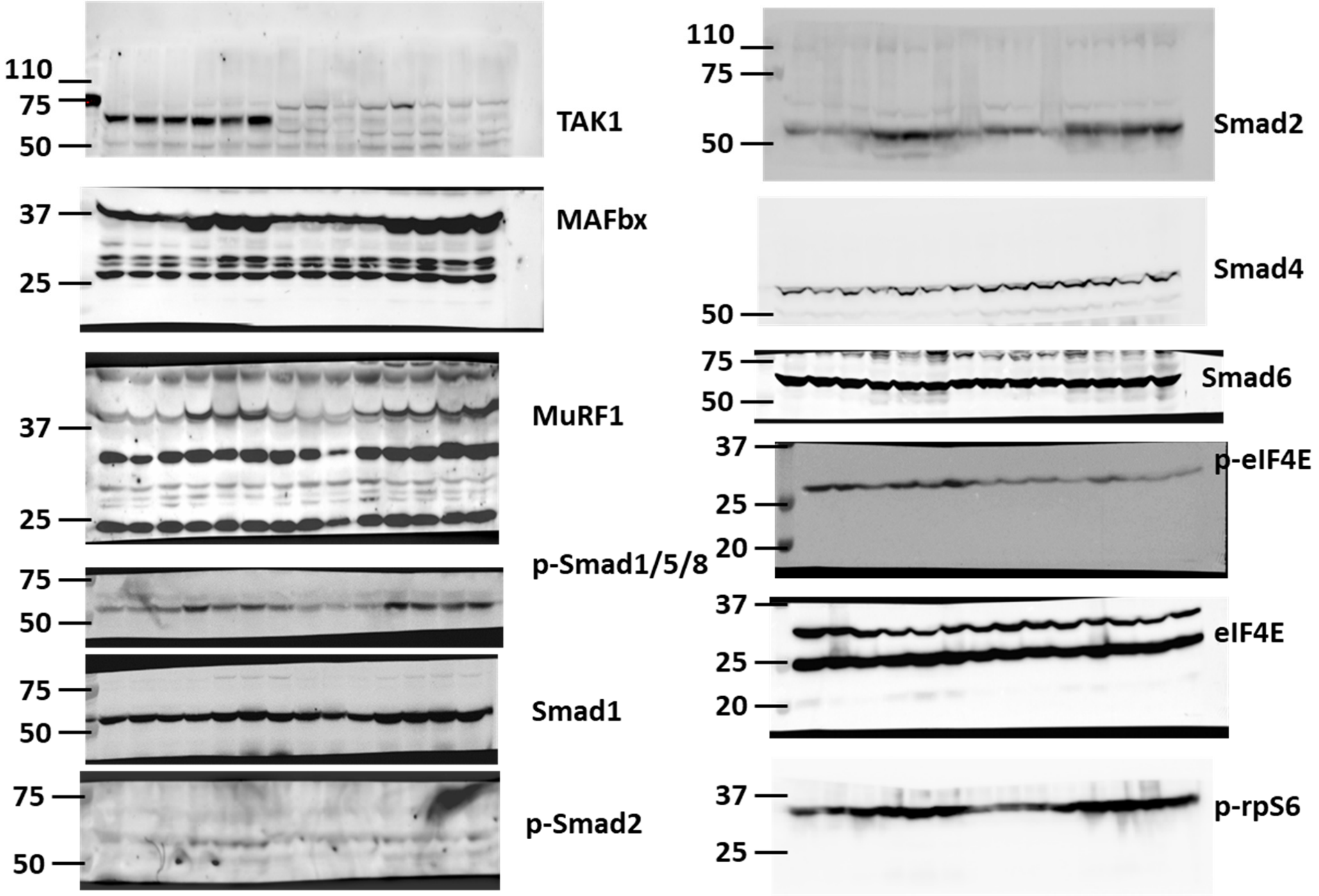

Supplementary Figure 9 contd.

Figure 6d contd.

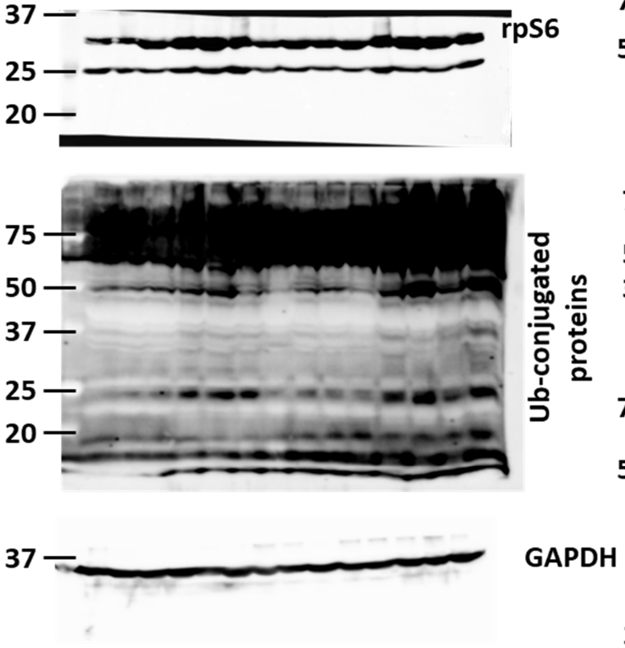

Figure 7b.

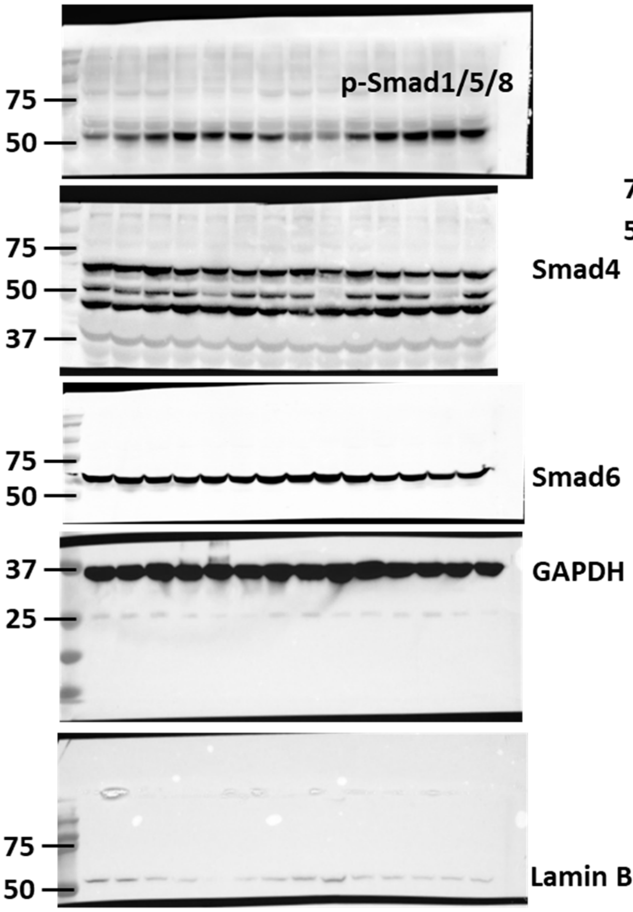

Figure 7a.

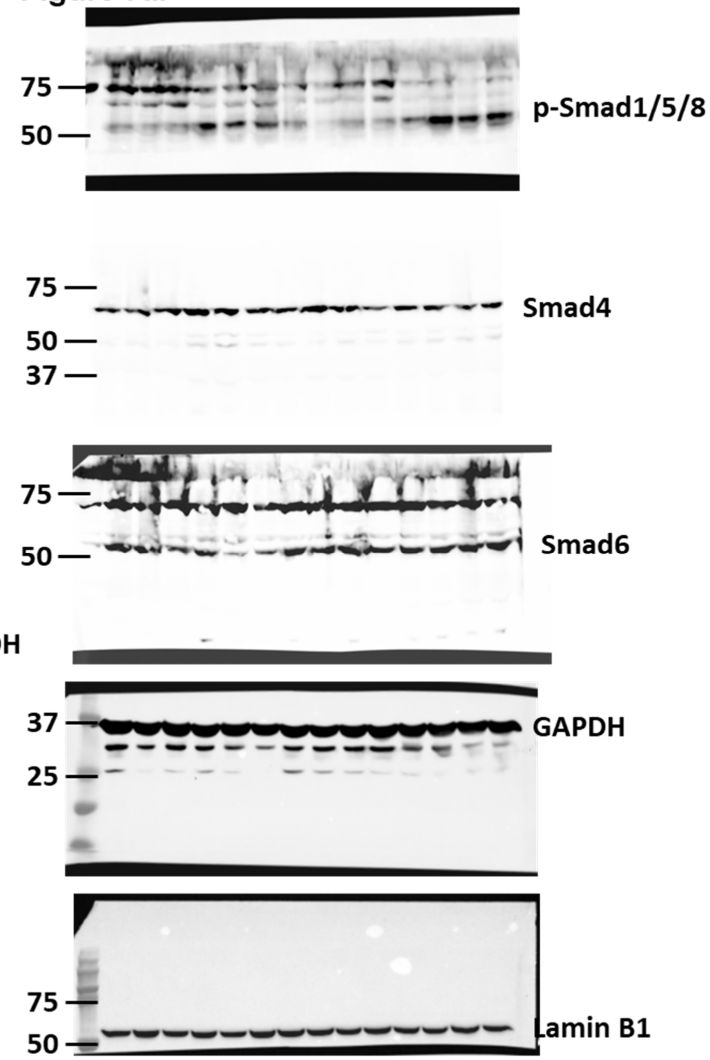

Supplementary Figure 9 contd.

Figure 8c.

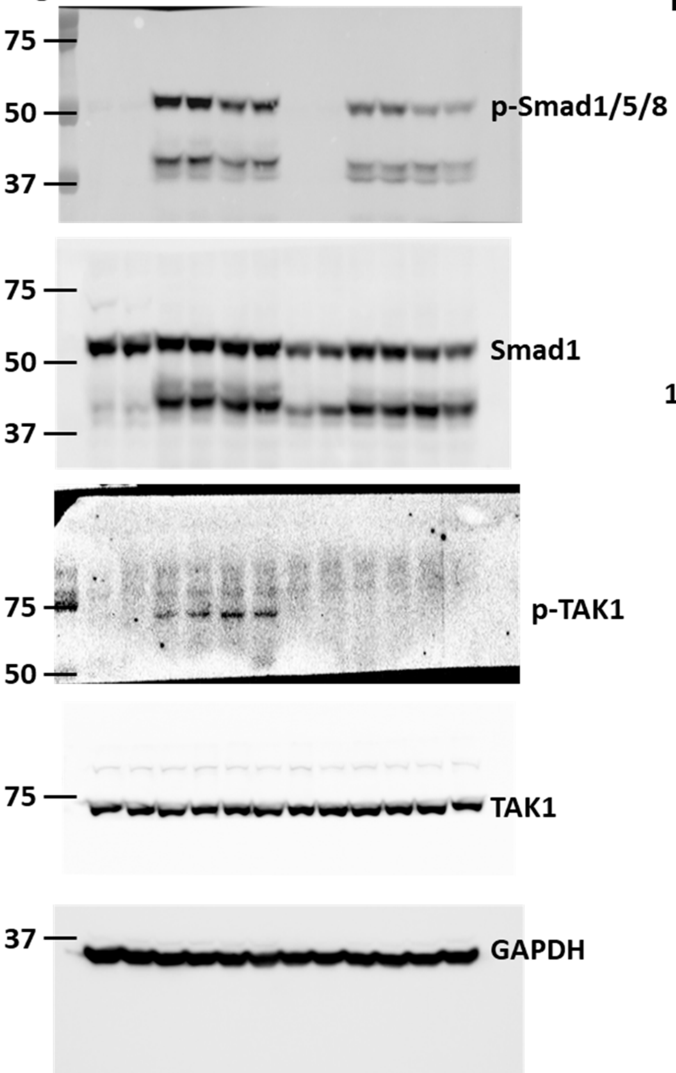

Figure 8f.

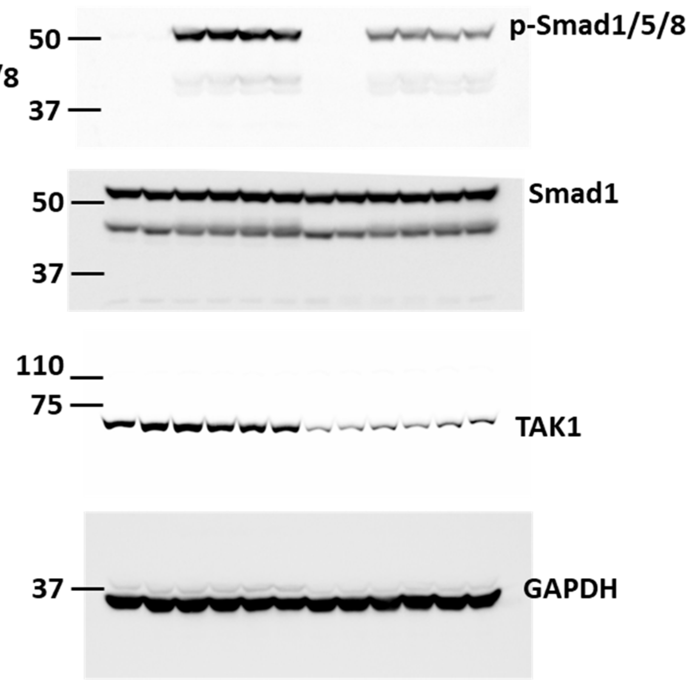

Supplementary Figure 9 contd.

Figure 9g.

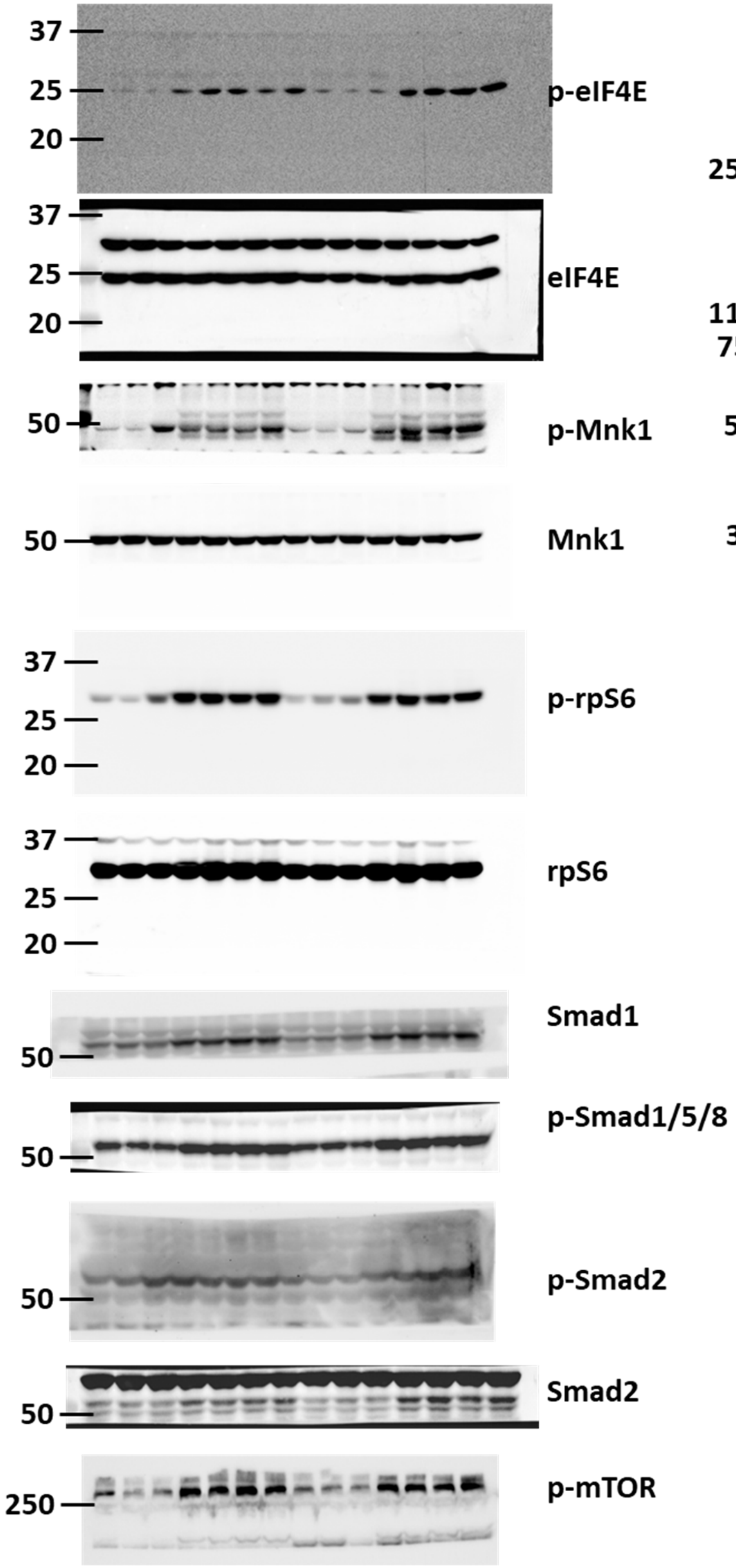

Figure 9g. Contd.

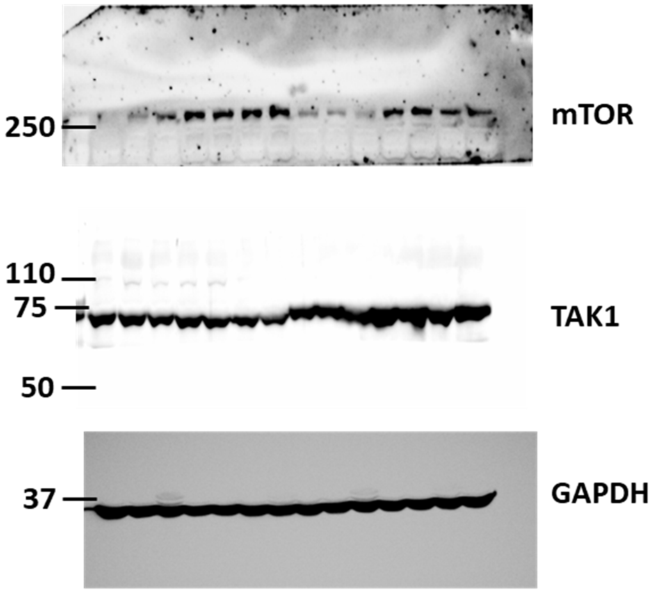

Figure S2d.

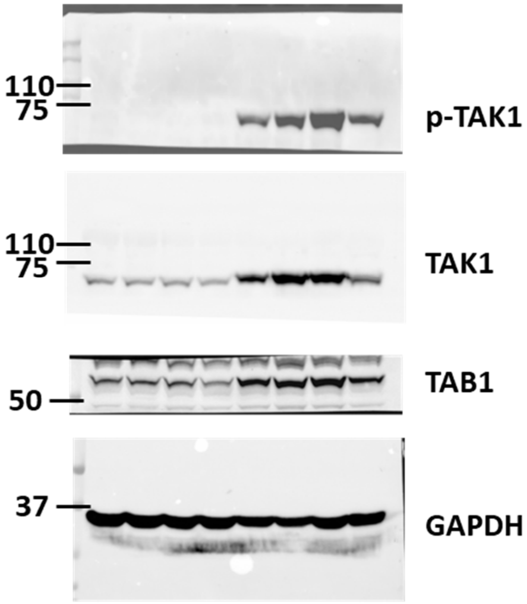

Supplementary Figure 9 contd.

Figure S3b.

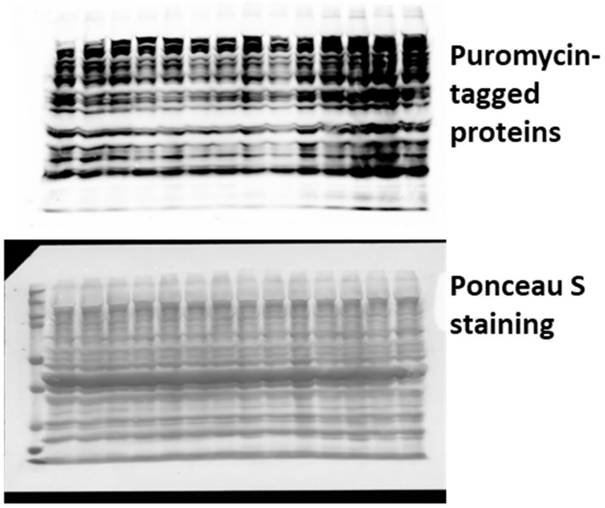

Figure S3c.

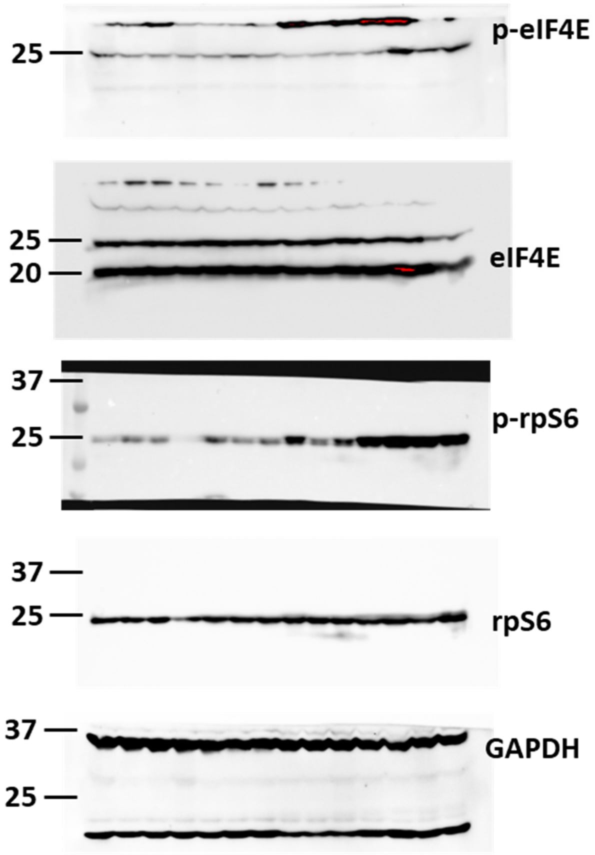

Figure S7a.

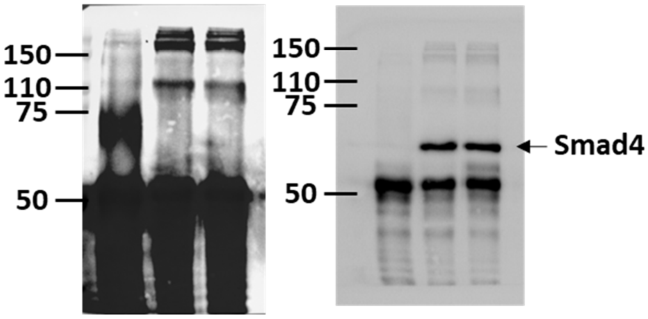

Supplementary Figure 9. Uncropped images of immunoblots used in the manuscript. Original uncropped images of the immunoblots as displayed in the main and supplemental figures of the manuscript are presented here. Yellow rectangle boxes were used to mark indicated bands where multiple bands appear in the same immunoblot. Molecular weight markers are presented on the left side of the immunoblots.

Supplementary Table 1. Antibodies used in the study.

**Antibody**	**Source and Catalog no.**
Monoclonal rabbit-anti-phospho-TAK1	Invitrogen #MA5-15073
Monoclonal rabbit-anti-total-TAK1	Cell Signaling Technology, # 5206
Monoclonal rabbit-anti-phospho-p38 MAPK	Cell Signaling Technology, # 4511
Polyclonal rabbit-anti-total-p38 MAPK	Cell Signaling Technology, # 9212
Monoclonal rabbit-anti-phospho-Smad1/5/9	Cell Signaling Technology # 13820
Monoclonal rabbit-anti-total-Smad1	Cell Signaling Technology # 6944
Monoclonal rabbit-anti-phospho-Smad2	Cell Signaling Technology # 3108
Monoclonal rabbit-anti-total-Smad2	Cell Signaling Technology # 5339
Polyclonal rabbit-anti-phospho-mTOR	Cell Signaling Technology # 2971
Polyclonal rabbit-anti-total-mTOR	Cell Signaling Technology # 2972
Monoclonal rabbit-anti-GAPDH	Cell Signaling Technology # 2118
Monoclonal rabbit-anti-phospho-p65 NF-κB	Cell Signaling Technology # 3033
Monoclonal rabbit-anti-total-p65 NF-κB	Cell Signaling Technology # 8242
Polyclonal rabbit-anti-phospho-p44/42 MAPK	Cell Signaling Technology # 9101
Monoclonal rabbit-anti-total-p44/42 MAPK	Cell Signaling Technology # 4695
Polyclonal rabbit-anti-Smad4	Cell Signaling Technology # 9515
Monoclonal rabbit-anti-phospho-Akt	Cell Signaling Technology # 4060
Polyclonal rabbit-anti-total-Akt	Cell Signaling Technology # 9517
Monoclonal rabbit-anti-phospho-AMPKα	Cell Signaling Technology # 2535
Polyclonal rabbit-anti-total AMPKα	Cell Signaling Technology # 2532
Polyclonal rabbit-anti-phospho-eIF4E	Cell Signaling Technology # 9741
Monoclonal rabbit-total-eIF4E	Cell Signaling Technology # 2067
Polyclonal rabbit-anti-phospho-eIF4B	Cell Signaling Technology # 3591
Polyclonal rabbit-total-eIF4B	Cell Signaling Technology # 3592
Monoclonal rabbit-eIF4A	Cell Signaling Technology # 2013
Monoclonal rabbit-eIF4H	Cell Signaling Technology # 3469
Monoclonal rabbit-anti-phospho-eIF2α	Cell Signaling Technology # 3398
Monoclonal rabbit-anti-total-eIF2α	Cell Signaling Technology # 5324
Polyclonal rabbit-anti-phospho-p70S6 Kinase	Cell Signaling Technology # 9208
Polyclonal rabbit-anti-total-p70S6 Kinase	Cell Signaling Technology # 9202
Monoclonal rabbit-anti-phospho-S6 Ribosomal Protein	Cell Signaling Technology # 4858
Monoclonal rabbit-anti-total-S6 Ribosomal Protein	Cell Signaling Technology # 2217
Polyclonal rabbit-anti-phopsho-Mnk1	Cell Signaling Technology # 2111
Monoclonal rabbit-anti-total-Mnk1	Cell Signaling Technology # 2195
Polyclonal rabbit-anti-phospho-p90RSK	Cell Signaling Technology # 9346
Monoclonal rabbit-anti-total-p90RSK	Cell Signaling Technology # 9355
Monoclonal rabbit-anti-FoxO1	Cell Signaling Technology # 2880

Monoclonal rabbit-anti-FoxO3a	Cell Signaling Technology # 12829
Polyclonal rabbit-anti-FoxO4	Cell Signaling Technology # 9472
Monoclonal rabbit-anti-HDAC4	Cell Signaling Technology # 7628
Monoclonal mouse-anti-myogenin	DSHB #F5D
Polyclonal rabbit-anti-α-Tubulin	Cell Signaling Technology # 2144
Polyclonal rabbit-anti-Smad6	Invitrogen # PA1-41026
Polyclonal rabbit-anti-MAFbx (Atrogin-1)	ECM Biosciences, AP2041
Polyclonal goat-anti-MuRF1	R&D Systems, AF5366
Monoclonal mouse-anti-Ubiquitin	Santa Cruz Biotechnology, sc-8017
Monoclonal rabbit-anti-Lamin B1	Cell Signaling Technology # 13435
Monoclonal mouse-anti-Puromycin	Millipore, MABE343
Polyclonal rabbit-anti-Laminin	Sigma, L9393
Polyclonal rabbit-anti-Dystrophin	Abcam, ab15277
Monoclonal mouse-anti-Myosin heavy chain	DSHB #MF 20
Monoclonal mouse-anti-SV2	DSHB #SV2
Monoclonal mouse-anti-(NF-M)	DSHB #2H3
Polyclonal goat-anti-mouse IgG Alexa Fluor 568	Invitrogen # A-11004
Polyclonal goat-anti-rabbit IgG Alexa Fluor 568	Invitrogen # A-11036

Supplementary Table 2. Sequence of the primers used for QRT-PCR in the study.

Gene Name	Forward primer (5'-3')	Reverse primer (5'-3')
Chrna1	AAG CTA CTG TGA GAT CAT CGT CAC	TGA CGA AGT GGT AGG TGA TGT CCA
Chrnb1	ACG GTC CAC AAC CAT GGC	CAT CAT CGC TCA CCC CAC
Agrn	CCT CAA CTT GGA CAC GAA GCT	AGG CCG ATG CCC ACA GA
Musk	TGA GAA CTG CCC CTT GGA ACT	GGG TCT ATC AGC AGG CAG CTT
Dok7	TCT CCC AGA CCC GAG TTC TG	TCT AGC TGC AGG GCT TCC
Hdac4	CAG ATG GAC TTT CTG GCC G	CTT GAG CTG CTG CAG CTT C
Myog	CAT CCA GTA CAT TGA GCG CCT A	GAG CAA ATG ATC TCC TGG GTT G
Bmpr1a	GAA AGA CCT GAT TGA CCA GTC C	CCC ATC CAT ACT TCT CCA TAG C
Bmpr1b	GAA TAC CAG CTT CCC TAT CAC G	TCT GCC TGA GAC ACT CAT CAC T
Bmpr2	TTG GAC TCA TCT ACT GGG AGG T	TGG ACA CAA GAA CCT GCA TAT C
Acvr1b	AAG CTG AGA GTT GGG AGA AG	GGG CTT TAG ACT TGG TCT GT
Acvr1c	AAC TTT GCC AAC AGC TAG TC	GGC AGA GAA GAA TGT ACA CC
Tgfbr2	CTT CAC TTC CGG GTC ATC AT	GCG ATG CTA TTC CTT GGT CT
Bmp4	GAC TTC GAG GCG ACA CTT CTA C	CAG ATG TTC TTC GTG ATG GAA A
Bmp7	AGC TTC GTC AAC CTA GTG GAA C	CTG GAG CAC CTG ATA GAC TGT G
Bmp8a	CCT ATT ACT GTG AGG GGG AGT G	TGA CAT TGT TGC TGC TGT CAT A
Bmp8b	TCC ACT TTG ACC TAA CCC AGA T	GTC AGA CTC CCT GTT GGA GTG
Bmp13	AAG ACT TAC TCC ATT GCC GAG A	TCG TCC AGT CCT CTG TCT ACA A
Bmp14	ATG CTG ACA GAA AGG GAG GTA A	GCA CTG ATG TCA AAC ACG TAC C
Bmp15	GAA AAT GGT GAG GCT GGT AAA G	TCG TAT GCT ACC TGG TTT GAT G
Tgfb1	CTG AAC CAA GGA GAC GGA ATA C	GGG CTG ATC CCG TTG ATT T
Tgfb2	GGC TTT CAT TTG GCT TGA GAT G	CTT CGG GTG AGA CCA CAA ATA G
Tgfb3	GTA CAT CTG CTC TAG GGA ATT GG	CCA GGC AGT GCA AGA TAT GA
Fst	AAC GTT GGG AGA GAG GAT GA	GAC AGG TTG AAA GTG TGC CT
Bmp2	GCA GCT TCC ATC ACG AAG A	GAT GTG AGA AAC TCG TCA CTG G
Bmp3	CGA ATG GAT TAT CTC TCC CAA G	AAC AAG ATG CTG AGT GAG GAC A
Bmp5	AGG AAT ACA CAA ACA GGG ATG C	CCA GCA GAT TTT ACA TTG ATG C
Bmp6	TCT TCA GAC TAC AAC GGC AGT G	ATC ACA GTA GTT GGC AGC GTA G
Bmp11	CTT GGA AGA GGA CGA GTA CCA C	CTG AAG TGG AAA TGA CAG CAG A
Bmp12	CAT GAT GTC GCT TTA CAG GAG	GAT ACG TCG AAC AGG AAG CTC T
Mstn	AAT CCA CCA CGG TGC TAA TG	TTA GTG CTG TGT GTG TGG AG
Inhba	AGA ACG GGT ATG TGG AGA TAG A	GAC TCG GCA AAG GTG ATG AT
Acvr2a	GTT ACA CCG AAG CCA CCC TA	AAC CAA ATC TTC CCC TTG CT
Acvr2b	TTA AGG ATC ACT GGC TGA AAC A	GGA TAC CCG CTC TTC TAC ACA G
Fbxo30	TCGTGGAATGGTAATCTTGC	CCTCCCGTTTCTCTATCACG
Tak1	GTCATCCAGCCCTAGTGTCAGAAT	TTCTTTGGAGTTTGGGCACG
β-actin	CAGGCATTGCTGACAGGATG	TGCTGATCCACATCTGCTGG
