# Supplementary material for: Supraphysiological activation of TAK1 promotes skeletal muscle growth and mitigates neurogenic atrophy
Source: Nat Commun. 2022 Apr 22;13:2201. doi: 10.1038/s41467-022-29752-0 (PMC9033787; doi:10.1038/s41467-022-29752-0)
Supplement: Supplementary file 2 — Reporting Summary [file 41467_2022_29752_MOESM2_ESM.pdf]

nature portfolio

Corresponding author(s): Ashok Kumar

Last updated by author(s): Feb 25, 2022

Reporting Summary

Nature Portfolio wishes to improve the reproducibility of the work that we publish. This form provides structure for consistency and transparency in reporting. For further information on Nature Portfolio policies, see our Editorial Policies and the Editorial Policy Checklist.

Statistics

For all statistical analyses, confirm that the following items are present in the figure legend, table legend, main text, or Methods section.

n/a	Confirmed	
☐	☒	The exact sample size (*n*) for each experimental group/condition, given as a discrete number and unit of measurement
☐	☒	A statement on whether measurements were taken from distinct samples or whether the same sample was measured repeatedly
☐	☒	The statistical test(s) used AND whether they are one- or two-sided<br>*Only common tests should be described solely by name; describe more complex techniques in the Methods section.*
☐	☒	A description of all covariates tested
☐	☒	A description of any assumptions or corrections, such as tests of normality and adjustment for multiple comparisons
☐	☒	A full description of the statistical parameters including central tendency (e.g. means) or other basic estimates (e.g. regression coefficient) AND variation (e.g. standard deviation) or associated estimates of uncertainty (e.g. confidence intervals)
☐	☒	For null hypothesis testing, the test statistic (e.g. *F*, *t*, *r*) with confidence intervals, effect sizes, degrees of freedom and *P* value noted<br>*Give P values as exact values whenever suitable.*
☒	☐	For Bayesian analysis, information on the choice of priors and Markov chain Monte Carlo settings
☒	☐	For hierarchical and complex designs, identification of the appropriate level for tests and full reporting of outcomes
☐	☒	Estimates of effect sizes (e.g. Cohen's *d*, Pearson's *r*), indicating how they were calculated

Our web collection on statistics for biologists contains articles on many of the points above.

Software and code

Policy information about availability of computer code

Data collection	No software was used for data collection.
Data analysis	NMJ-morph - ImageJ, Graphpad Prism 9, NIS Elements BR 3.10 software (Nikon)

For manuscripts utilizing custom algorithms or software that are central to the research but not yet described in published literature, software must be made available to editors and reviewers. We strongly encourage code deposition in a community repository (e.g. GitHub). See the Nature Portfolio guidelines for submitting code & software for further information.

Data

Policy information about availability of data

All manuscripts must include a data availability statement. This statement should provide the following information, where applicable:

- Accession codes, unique identifiers, or web links for publicly available datasets
- A description of any restrictions on data availability
- For clinical datasets or third party data, please ensure that the statement adheres to our policy

The authors confirm that the data supporting the findings of this study are available in the supplementary files and source data. All relevant data related to this manuscript are available from the authors upon reasonable request.

Field-specific reporting

Please select the one below that is the best fit for your research. If you are not sure, read the appropriate sections before making your selection.

[x] Life sciences [ ] Behavioural & social sciences [ ] Ecological, evolutionary & environmental sciences

For a reference copy of the document with all sections, see nature.com/documents/nr-reporting-summary-flat.pdf

Life sciences study design

All studies must disclose on these points even when the disclosure is negative.

Sample size	Described in Methods section as well. We calculated sample size using size power analysis methods for a priori determination based on the s.d. and the effect size was previously obtained using the experimental procedures employed in the study. For animal studies, we estimated sample size from expected number of Tak1mKO mice and littermate Tak1fl/fl controls. We calculated the minimal sample size for each group as eight animals. Considering a likely drop-off effect of 10%, we set sample size of each group of six mice. For some experiments, three to four animals were found sufficient to obtain statistical differences.
Data exclusions	The exclusion criteria for animals were established in consultation with IACUC members and experimental outcomes. In case of death, skin injury, sickness or weight loss of >10%, the animal was excluded from analysis. Muscle tissue samples were not used for analysis in cases such as freeze artefacts on histological section or failure in extraction of RNA or protein of suitable quality and quantity.
Replication	Experiments were replicated/performed in three batches. We found consistent results in all the replicates.
Randomization	Animals from different breeding cages were included by random allocation to the different experimental groups.
Blinding	Animal experiments were blinded using number codes till the final data analyses were performed.

Reporting for specific materials, systems and methods

We require information from authors about some types of materials, experimental systems and methods used in many studies. Here, indicate whether each material, system or method listed is relevant to your study. If you are not sure if a list item applies to your research, read the appropriate section before selecting a response.

Materials & experimental systems

n/a	Involved in the study	
[ ]	[x]	Antibodies
[x]	[ ]	Eukaryotic cell lines
[x]	[ ]	Palaeontology and archaeology
[ ]	[x]	Animals and other organisms
[x]	[ ]	Human research participants
[x]	[ ]	Clinical data
[x]	[ ]	Dual use research of concern

Methods

n/a	Involved in the study	
[x]	[ ]	ChIP-seq
[x]	[ ]	Flow cytometry
[x]	[ ]	MRI-based neuroimaging

Antibodies

Antibodies used

Antibody Source and Catalog no. Dilutions
Monoclonal rabbit-anti-phospho-TAK1, Invitrogen #MA5-15073,1:500
Monoclonal rabbit-anti-total-TAK1, Cell Signaling Technology, # 5206, 1:500
Monoclonal rabbit-anti-phospho-p38 MAPK, Cell Signaling Technology, # 4511, 1:500
Polyclonal rabbit-anti-total-p38 MAPK, Cell Signaling Technology, # 9212, 1:500
Monoclonal rabbit-anti-phospho-Smad1/5/9, Cell Signaling Technology # 13820, 1:500
Monoclonal rabbit-anti-total-Smad1, Cell Signaling Technology # 6944, 1:500
Monoclonal rabbit-anti-phospho-Smad2, Cell Signaling Technology # 3108, 1:500
Monoclonal rabbit-anti-total-Smad2, Cell Signaling Technology # 5339, 1:500
Polyclonal rabbit-anti-phospho-mTOR, Cell Signaling Technology # 2971, 1:500
Polyclonal rabbit-anti-total-mTOR, Cell Signaling Technology # 2972, 1:500
Monoclonal rabbit-anti-GAPDH, Cell Signaling Technology # 2118, 1:1000
Monoclonal rabbit-anti-phospho-p65 NF-κB, Cell Signaling Technology # 3033, 1:500
Monoclonal rabbit-anti-total-p65 NF-κB, Cell Signaling Technology # 8242, 1:500
Polyclonal rabbit-anti-phospho-p44/42 MAPK, Cell Signaling Technology # 9101, 1:500
Monoclonal rabbit-anti-total-p44/42 MAPK, Cell Signaling Technology # 4695, 1:500
Polyclonal rabbit-anti-Smad4, Cell Signaling Technology # 9515, 1:500

	Monoclonal rabbit-anti-phospho-Akt, Cell Signaling Technology # 4060, 1:500<br>Polyclonal rabbit-anti-total-Akt, Cell Signaling Technology # 9517, 1:500<br>Monoclonal rabbit-anti-phospho-AMPKα, Cell Signaling Technology # 2535, 1:500<br>Polyclonal rabbit-anti-total AMPKα, Cell Signaling Technology # 2532, 1:500<br>Polyclonal rabbit-anti-phospho-eIF4E, Cell Signaling Technology # 9741, 1:500<br>Monoclonal rabbit-total-eIF4E, Cell Signaling Technology # 2067, 1:500<br>Polyclonal rabbit-anti-phospho-eIF4B, Cell Signaling Technology # 3591, 1:500<br>Polyclonal rabbit-total-eIF4B, Cell Signaling Technology # 3592, 1:500<br>Monoclonal rabbit-eIF4A, Cell Signaling Technology # 2013, 1:500<br>Monoclonal rabbit-eIF4H, Cell Signaling Technology # 3469 , 1:500<br>Monoclonal rabbit-anti-phospho-eIF2α , Cell Signaling Technology # 3398, 1:500<br>Monoclonal rabbit-anti-total-eIF2α , Cell Signaling Technology # 5324, 1:500<br>Polyclonal rabbit-anti-phospho-p70S6 Kinase, Cell Signaling Technology # 9208, 1:500<br>Polyclonal rabbit-anti-total-p70S6 Kinase , Cell Signaling Technology # 9202, 1:500<br>Monoclonal rabbit-anti-phospho-S6 Ribosomal Protein , Cell Signaling Technology # 4858, 1:500<br>Monoclonal rabbit-anti-total-S6 Ribosomal Protein , Cell Signaling Technology # 2217, 1:500<br>Polyclonal rabbit-anti-phopsho-Mnk1 , Cell Signaling Technology # 2111, 1:500<br>Monoclonal rabbit-anti-total-Mnk1 , Cell Signaling Technology # 2195, 1:500<br>Polyclonal rabbit-anti-phospho-p90RSK, Cell Signaling Technology # 9346, 1:500<br>Monoclonal rabbit-anti-total-p90RSK, Cell Signaling Technology # 9355, 1:500<br>Monoclonal rabbit-anti-FoxO1, Cell Signaling Technology # 2880, 1:500<br>Monoclonal rabbit-anti-FoxO3a, Cell Signaling Technology # 12829, 1:500<br>Polyclonal rabbit-anti-FoxO4 Cell, Signaling Technology # 9472, 1:500<br>Monoclonal rabbit-anti-HDAC4 ,Cell Signaling Technology # 7628, 1:500<br>Monoclonal mouse-anti-myogenin ,DSHB #F5D, 1:500<br>Polyclonal rabbit-anti-α-Tubulin , Cell Signaling Technology # 2144, 1:500<br>Polyclonal rabbit-anti-Smad6, Invitrogen # PA1-41026, 1:500<br>Polyclonal rabbit-anti-MAFbx, (Atrogin-1) ECM Biosciences, AP2041, 1:500<br>Polyclonal goat-anti-MuRF1 ,R&D Systems, AF5366, 1:500<br>Monoclonal mouse-anti-Ubiquitin, Santa Cruz Biotechnology, sc-8017, 1:1000<br>Monoclonal rabbit-anti-Lamin B1, Cell Signaling Technology # 13435, 1:500<br>Monoclonal mouse-anti-Puromycin , Millipore, MABE343, 1:1000<br>Polyclonal rabbit-anti-Laminin, Sigma, L9393, 1:200<br>Polyclonal rabbit-anti-Dystrophin Abcam, ab15277, 1:200<br>Monoclonal mouse-anti-Myosin heavy chain, DSHB #MF 20, 1:200<br>Monoclonal mouse-anti-SV2 ,DSHB #SV2, 1:200<br>Monoclonal mouse-anti-(NF-M), DSHB #2H3, 1:200<br>Polyclonal goat-anti-mouse IgG Alexa Fluor 568, Invitrogen # A-11004, 1:500<br>Polyclonal goat-anti-rabbit IgG Alexa Fluor 568, Invitrogen # A-11036, 1:500
Validation	A per the manufacturer's product information the antibodies has been validated for the intended use.

Animals and other organisms

Policy information about studies involving animals; ARRIVE guidelines recommended for reporting animal research

Laboratory animals	Mice, C57BL6, 2-4 months old, both male and female were were housed in a 12-h light-dark cycle and given water and food ad libitum. Temperatures of 65-75°F (~18-23°C) with 40-60% humidity was maintained.
Wild animals	Study did not use wild animals.
Field-collected samples	Study did not use Field-collected samples.
Ethics oversight	All the animals were handled according to approved institutional animal care and use committee (IACUC) protocols (protocol # PR201900043) of the University of Houston.

Note that full information on the approval of the study protocol must also be provided in the manuscript.
